# Antiaging and Antioxidant Bioactivities of Asteraceae Plant Fractions on the Cellular Functions of the Yeast *Schizosaccharomyces pombe*

**DOI:** 10.1155/2021/2119634

**Published:** 2021-09-18

**Authors:** Rika Indri Astuti, Muhammad Eka Prastya, Irmanida Batubara, Eka Budiarti, Aulia Ilmiyawati

**Affiliations:** ^1^Department of Biology, Faculty of Mathematics and Natural Sciences, IPB University, IPB Dramaga Campus, Bogor, West Java 16680, Indonesia; ^2^Tropical Biopharmaca Research Center, IPB University, Jl. Taman Kencana No. 3, Bogor, West Java 16128, Indonesia; ^3^Research Center for Chemistry, Indonesian Institute of Sciences (LIPI), National Research and Innovation Agency (BRIN), Kawasan PUSPITEK, Serpong, Tangerang Selatan, Banten 15314, Indonesia; ^4^Department of Chemistry, Faculty of Mathematics and Natural Sciences, IPB University, IPB Dramaga Campus, Bogor, West Java 16680, Indonesia

## Abstract

Research on antioxidants has been gaining worldwide attention because of their essential applications for medicinal purposes. In this study, we conducted bioprospecting of six Asteraceae plants as the source of antiaging and antioxidant agents. Water and chloroform fractions from *Ageratum conyzoides* L., *Dichrocephala integrifolia* (L.f.) Kuntze, *Galinsoga parviflora* (Cav.), *Mikania micrantha* Kunth, *Sphagneticola trilobata* (L.) Pruski, and *Synedrella nodiflora* L. were collected and assayed for their in vitro antioxidant activities and potential antiaging properties using the yeast *Schizosaccharomyces pombe* as the model organism. Based on the in vitro assay, the water fractions of *S. trilobata* showed a strong antioxidant activity. Interestingly, all treatment solutions promoted the stress tolerance phenotype of *S. pombe* to strong H_2_O_2_-induced oxidative stress conditions. Moreover, compared with the treatments without plant extract/fraction, all extract and fraction treatments, except the chloroform fractions of *A. conyzoides*, promoted yeast cell longevity. Strong induction of mitochondria activity was found following the treatments with the extracts and fractions of *S. nodiflora*, *D. integrifolia*, and *M. micrantha* and likely mimicked the calorie restriction-induced lifespan. Interestingly, *S. nodiflora* water fractions significantly upregulated the mRNA transcripts of the Pap1-mediated core environmental stress response, namely, ctt1 gene in *S. pombe*. These data indicated that the fractions of Asteraceae plants had potential antioxidant and antiaging activities through various cellular modulations. *S. nodiflora* water fraction has been shown to have antioxidant and antiaging activities in *S. pombe*, by modulating stress tolerance response, inducing mitochondrial activity, and increasing the ctt1 gene expression. Compounds analysis identified that *S. nodiflora* water fraction contained some primarily compounds including oxyphyllacinol, valine, and sugiol.

## 1. Introduction

Free radical accumulation has been known to induce oxidative stress conditions, which further damage cellular functions [1, 2]. Through the activation of enzymatic and nonenzymatic antioxidants, the human body has complex defense systems against these damaging effects of free radicals [3, 4]. As the body ages or after intensive exposure to exogenous free radical sources, the cellular defense systems may be compromised and cause severe oxidative stress conditions, which often culminate in the development of degenerative diseases [5]. Exogenous antioxidants, which are also known as dietary antioxidants, are often used to reduce the damaging effects of free radicals. Therefore, the intake of exogenous antioxidants may compensate for the inadequacy of cellular antioxidant activity in combating oxidative stress conditions [6].

Plants have been known as the major sources of antioxidant compounds, most of which are phenolic [7, 8]. Among the plant families, Asteraceae is the second largest family and is the largest family of flowering plants in the kingdom Plantae. Therefore, exploration of plants that belong to the Asteraceae family is widely open, especially for those considered as weed. In fact, weed has been the most subjected plant for intensive control in farm fields; however, their value for bioprospecting may not have been properly explored. Previous studies have revealed the medicinal value of Asteraceae plants in vitro [9–12] and suggested the potential use of plant extract as dietary antioxidants. However, until today, analyses of the antioxidant activities of Asteraceae-derived extracts in modulating cellular systems have been very limited. Such information is essential for the comprehensive evaluation of the antioxidant properties of the extracts, especially in revealing the mechanism of actions at the cellular level, and the application of these extracts as dietary antioxidants for daily use.

In this study, we analyzed the potential antioxidant activity of the weed plant extracts of *Ageratum conyzoides* L., *Dichrocephala integrifolia* (L.f.) Kuntze, *Galinsoga parviflora* (Cav.), *Mikania micrantha* Kunth, *Sphagneticola trilobata* L. Pruski, and *Synedrella nodiflora* L. The effect of those extracts at the cellular level was evaluated using the yeast *Schizosaccharomyces pombe* as the model organism. Our previous study has already reported the antioxidant activity of the water extract of each plant sample [13]. Therefore, in this study, we further determined the potential antioxidant properties of the water and chloroform fractions, as well as the water extract of each plant, based on the capability of promoting yeast longevity. Oxidative stress has been known to be a major factor in cellular aging. Therefore, the application of exogenous antioxidants may inhibit cellular aging and promote longevity [14]. The molecular pathways of cellular aging between *S. pombe* and the multicellular eukaryotic organisms are homologous and involve the activities of the sirtuin [15, 16] and mitogen-activated protein kinase genes [17, 18], autophagy [19, 20], and mitochondria [21–23]. In addition, calorie restriction has also been implied as exogenous treatment that induces the complex longevity pathway which was conserved from yeast to human [23, 24]. Therefore, in this research, we aimed to determine the mechanism of actions of particular plant extracts by evaluating their regulatory functions on the activities of the mitochondria and the oxidative stress response genes.

## 2. Materials and Methods

### 2.1. Samples

Six species of Asteraceae that easily grow in Indonesia were collected from the Conservation and Cultivation Unit of Tropical Biopharmaca Research Center Bogor, West Java, Indonesia (GPS location: 6°35′17.8″S 106°48′05.4″E). Each species was determined by a botanist in the Indonesian Institute of Sciences (LIPI) Biology at Cibinong, Jakarta, Indonesia. Voucher specimens of the samples were deposited in the Tropical Biopharmaca Research Center, IPB University, Indonesia. The list of samples and voucher specimen numbers is shown in Table 1. For this study, the aerial plant parts, including twigs and leaves, were used.

### 2.2. Yeast Strain and Growth Conditions

We used the yeast strain of *S. pombe* ARC039 (h-leu1-32 ura4-294) to investigate the antiaging effects of the extracts at the cellular level [25]. For the viability assay, the yeast cells were grown in Yeast Extract with Supplements (YES) medium with 3% glucose and in Edinburgh Minimal Medium (EMM) with 0.5% glucose as the negative and positive controls, respectively [26].

### 2.3. Extraction and Fractionation

All samples were extracted with water (1 g dried sample to 10 mL water) at room temperature for 24 hours. Thereafter, the filtrate was separated from the residue and was concentrated by a rotary evaporator to get the crude extract. Then, the crude extract was separated by liquid-liquid extraction using chloroform, in order to get the water and chloroform fractions. Each fraction was concentrated using a rotary evaporator.

### 2.4. Antioxidant Assay

The antioxidant activities of the fractions were determined using the radicals of 2,2′-diphenylpicryl hydrazyl (DPPH, Sigma-Aldrich) and 2,2′-*azino-bis*(3-ethylbenzothiazoline-6-sulfonic acid) (ABTS, Carbosynth Ltd., UK). The anti-DPPH scavenging activity was determined using spectrophotometry assay, as described elsewhere [27]. The fractions (100–1500 *µ*g/mL) were diluted with ethanol (96%), reacted with DPPH, and incubated for 30 minutes. Thereafter, the absorbance of the solution was determined at 514 nm. The anti-ABTS activity was determined using a spectrophotometric method [27]. Briefly, 7 mM ABTS was oxidized by potassium peroxide sulfate to produce the ABTS radical, which was then reacted with a fraction solution (10 mg/mL in 96% ethanol). The absorbance of the solution was measured at 734 nm. Both DPPH and ABTS assays were conducted using a multiscan ELISA microtiter plate reader (EPOC Biotech, USA). The ability of the fractions to scavenge DPPH and ABTS radicals was calculated as % inhibition by the same following equation:

(1) $$\begin{array}{c} \%\text{ inhibition}=1-\left\{\frac{\left(\text{Abs}.\text{ sample}-\text{Abs}.\text{ blank}\right)}{\text{Abs}.\text{ control}-\text{Abs}.\text{ blank}}\right\}\times 100. \end{array}$$

Here, Abs.sample is the absorbance of DPPH/ABTS radicals + fractions or positive control (ascorbic acid/Trolox); Abs.control and Abs.blank are the absorbance of the DPPH/ABTS radicals + ethanol and the absorbance of ethanol, respectively. As for DPPH activity, the results are expressed as the inhibitory concentration of 50% (IC_50_), whereas ABTS activity is reported in mg Trolox equivalent (TE)/g sample.

### 2.5. Oxidative Stress Tolerance Assay

The experiment was carried out to evaluate the effect of the plant extracts/fractions in promoting cell viability against oxidative stress [28]. The yeast cells were inoculated in YES liquid medium for the treatment culture, with an initial OD_600_ of 0.05. Simultaneously, different concentrations of the extracts/fractions (at concentrations ranging from 1 to 5 times the IC_50_ value based on the DPPH assay) dissolved in 99% DMSO (at a final concentration of 5%) were added to the treatment culture. After incubation for 7 and 11 days with centrifugation at 120 rpm at 30°C, the yeast cells were streaked onto the plates with YES medium containing 5 mM H_2_O_2_. After three days of incubation at 30°C, cell viability was observed based on the density of the colonies on the agar plate. In this assay, the yeast cells cultured in YES and EMM media, which were both supplemented with 99% DMSO (at a final concentration of 5%), were used as negative and positive controls, respectively.

### 2.6. Aging Assay

Aging assay was conducted based on a previous study [29]. The yeast cells were grown in YES liquid medium containing the plant extracts/fractions. The applied concentration of the extracts/fractions was based on the oxidative stress tolerance assay (i.e., 1–5 times concentrations of IC_50_, based on the DPPH activity). Briefly, the yeast culture was prepared as described above in the oxidative stress tolerance assay. The yeast cells were harvested on days 7 and 11 and were serially diluted in sterile water at an initial OD_600_ of 1. Next, 3 *µ*L of each dilution was spotted onto the YES agar plates and incubated for three days at 30°C. Cell viability was evaluated qualitatively.

For further evaluation, the number of cells in the day 11 culture was quantified based on the plate count assay [30]. Day 11 cultures were harvested and serially diluted using sterile water, as described previously. Thereafter, about 100 *µ*L of the culture was spread onto the YES agar plate and incubated for three days at 30°C. The number of cells was then enumerated. The yeast cells that grew in the EMM and YES media, which were both supplemented with 99% DMSO (at a final concentration of 5%), were used as the positive and negative controls, respectively.

### 2.7. Mitochondrial Activity Assay

The mitochondrial activity and the mitochondrial membrane potential of the yeast cells were analyzed using a fluorescent probe (Rhodamine B; Sigma-Aldrich, USA) [31]. The yeast cells were cultured in YES medium supplemented with the plant extracts/fractions (with the concentration from 1 to 5 times of IC_50_, following the DPPH activity) at an initial OD_600_ of 0.05 to the log phase. Further, the yeast cells were harvested and washed with phosphate-buffered saline (PBS), which had a pH of 7.4, prior to suspension with Rhodamine B at a final concentration of 100 nM on PBS. After incubation for 30 minutes, the yeast cells were washed, and mitochondrial activity was observed on a fluorescent microscope (Olympus BX51).

### 2.8. Gene Expression Analysis Using Quantitative Reverse Transcriptase-Polymerase Chain Reaction

The best concentrations of plant extracts/fractions (*S. nodiflora*: water extract: 8000 ppm, water fraction: 1794 ppm, and chloroform fraction: 3885 ppm) dissolved in 99% DMSO (at a final concentration of 5%) were supplemented onto the YES liquid medium after the addition of the yeast inoculum at an initial OD_600_ of 0.05. Notably, 99% DMSO at a final concentration of 5% was used as the control for these experiments. After incubating the particular treatment for 24 hours, RNA was extracted from the yeast cells using the RNeasy Mini Kit (Qiagen, USA). Reverse transcription was done using iScript™ cDNA Synthesis Kit (Bio-Rad, USA), with iScript reverse transcriptase and 500 ng of total RNA. Quantitative reverse transcriptase-polymerase chain reaction was performed using Applied Biosystems StepOnePlus™ Instrument and Thunderbird SYBR qPCR master mix (Toyobo, Japan) as the fluorescent reporter. The thermal cycling parameters were as follows: 40 cycles, 95°C for 15 seconds, 55°C for 30 seconds, and 72°C for 30 seconds. The list of primers from the target genes, including act1 (housekeeping gene), pap1, ctt1, and sod2, is shown in Table 2. To measure the relative expression level, the cycle threshold (Ct) values of all target genes were normalized to the Ct of act1.

### 2.9. Liquid Chromatography Quadrupole-Mass Spectrometry (LC-MS/MS)

The selected fractions were analyzed using Xevo G2-XS QTof (Quadrupole Time-of-Flight) mass spectrometry instrument (Waters, USA) via an electron spray interface (ESI). Chromatographic separation conditions were performed using an LC system in the form of Ultra Performance Liquid Chromatography (UPLC)/QTof MS analytical system (Waters). Separation was achieved by stepwise gradients from 95% A (0.1% formic acid + distilled water) and 5% B (acetonitrile + 0.1% formic acid) to 5% A and 95% B for 16 minutes. The flow rate of the desolvation gas was set to 1000 L/h, for cone gas it was set to 50 L/h, and the source temperature was fixed to 120°C. The capillary voltage and cone voltage were set to 2.0 and 30 kV, respectively. The mass spectrometry was determined using the type of electrospray ionization (ES) Xevo G2-S QTof (Waters) with Quadrupole Time-of-Flight mass spectrometry in positive ion mode. Moreover, the accurate mass and composition for the precursor ions and fragment ions were calculated and identified using the UNIFI software library incorporated in the instrument.

### 2.10. Statistical Analyses

All data were represented as mean ± SEMs (*n* = 3). The significant differences among the groups in all experiments were determined using one-way analysis of variance with 95% confidence level, followed by multiple Duncan test ranges. A *p*-value of less than 0.05 was considered statistically significant.

## 3. Results and Discussion

### 3.1. Antioxidant Activity of Plant Extracts and Fractions In Vitro

**Figure 1:**
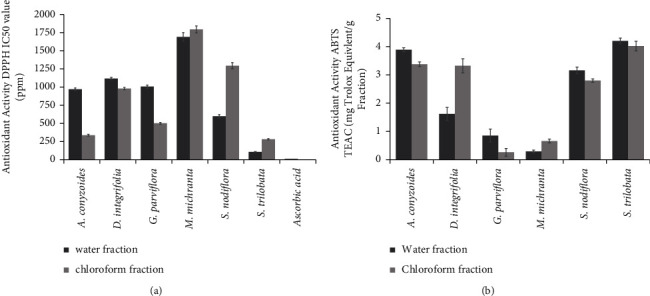
Antioxidant activity of fractions tested in this study. (a) DPPH activity expressed in IC_50_ value (the ability of fractions to scavenge DPPH radicals at 50%). (b) ABTS method depicted in TEAC (Trolox equivalent antioxidant activity) mg Trolox equivalent/g fraction.

In the present study, we used water as for particular solvent to extract the primary compounds of all samples. Of note, water has been used routinely to extract active compounds from plants in the early step of making Indonesian herbal medicine, namely, “Jamu,” traditionally and industrially. We suggested that the corresponding methods used in this study could obtain the various active compounds and thus would provide new valuable insight for developing “Jamu” derived from Asteraceae plant extract. In the initial study and on the basis of the in vitro antioxidant assays using DPPH and ABTS radicals, the water fraction of *S. trilobata* showed the highest antioxidant activity (Figures 1(a) and 1(b)). As shown in Figure 1(a), the IC_50_ value of *S. trilobata* water fraction (108.09 ppm) is the lowest compared to others which indicated strong activity to scavenge DPPH radicals. Of note, IC_50_ was defined as the concentration that could inhibit a reaction by about 50%. The most active DPPH samples were those with the lowest IC_50_ values. On the other hand, the anti-ABTS samples were measured as the Trolox equivalent antioxidant activity (TEAC, mg Trolox equivalent/g). The most active ABTS sample was the sample with the highest value of TEAC. Together, *S. nodiflora* water fraction exhibited the highest value of 4.20 mg Trolox/g fraction in the ABTS assay (Figure 1(b)). Nevertheless, the activity of the particular water fraction was not as good as that of pure ascorbic acid as the positive control.

### 3.2. Antioxidant Activity of the Plant Extracts and Fractions on Yeast Cell Viability

To determine the optimum concentrations of the plant extract and fractions for regulating the oxidative stress response in yeast cells, we tested the effects of various concentrations of the plant extract and fractions on the cell viability of yeast in H_2_O_2_-induced oxidative stress conditions. The concentrations of the plant extracts/fractions used in this assay were determined starting from 1 × IC_50_ value of DPPH activity followed by 2 up to 5 times IC_50_. Based on our results, treatment with all plant extracts and fractions (except A. *conyzoides* chloroform fraction) enhanced the stress tolerance phenotype of the yeast *S. pombe*, compared with the effects seen without treatment (control) (Table 3). In addition, those plant extracts and fractions treatment resulted in higher cell viability than that of calorie restriction treatment (Table 3). Calorie restriction treatment has been known to trigger a reduction in cellular damage induced by reactive oxygen species [32]. The effect of extracts/fractions treatments was indicated by the viability of the remaining cells that were still viable on the spot assay under the oxidative stress condition as shown in the additional figure in Table 3. This reveals the potential activity of plant extracts treatments in mimicking calorie restriction treatment even at high-calorie nutrition (3% glucose in YES medium) used in this study.

Among the extract and fraction concentrations, the water extract of *D. integrifolia* and the water fraction of *G. parviflora* highly promoted cell viability against oxidative stress. However, such phenotype was observed following high concentrations of the extracts/fractions treatment (5x times of the IC_50_). On the other hand, a lower value which indicated stronger activity of 3x times from the IC_50_ concentrations of both *S. nodiflora* water and chloroform fractions highly induced an oxidative stress tolerance phenotype in the yeast *S. pombe,* thereby suggesting the potential application as an exogenous antioxidant.

Our data indicated that the plant extracts/fractions induced the capability of yeast cells to cope with strong oxidative stress conditions. Previous studies revealed the potential of various extracts of plants, including rosella and clove, in promoting yeast viability in oxidative stress conditions [25, 33]. The particular extract with such antioxidant activities may be developed as an antiaging agent. Indeed, a comprehensive review presented the antioxidant properties of various plants, which were applied to promote human skin cell longevity [34]. Therefore, in this study, we analyzed and determined the potential antiaging properties of the Asteraceae plant extracts using yeast as the model organism.

### 3.3. Antiaging Activity of Plant Extracts and Fractions

**Figure 2:**
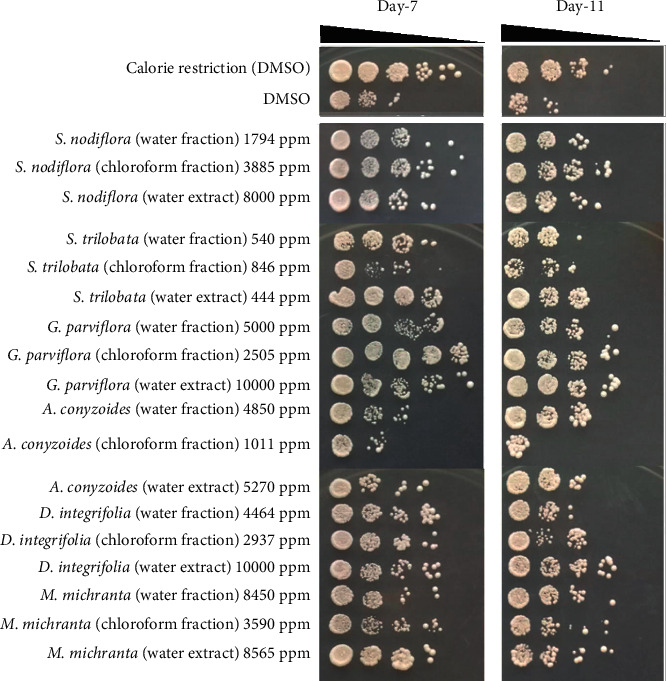
Effect of plant extract and fractions on yeast cells viability during stationary phase (chronological aging). Yeast cells were grown in a YES medium containing an optimum concentration of plant extract/fractions as observed previously. Yeast cells grown in EMM medium supplemented with DMSO were designated as calorie restriction for control positive treatment, while yeast cells grown in YES medium supplemented with DMSO only without any extract/fractions addition were used as control. Each culture treatment was then incubated for 7 and 11 days and subjected for spot assay on YES agar. Plates were incubated for three days at 30°C.

Based on spot chronological lifespan assays, yeast cell viability can be observed from the spotted assays that emerge on the surface of the plate medium as shown in Figure 2. More spotted assays in the horizontal direction on day 11 indicated high yeast cell viability after 11 days of treatments. All treatments with the extracts and fractions, except those with the chloroform fractions of *A. conyzoides*, were shown to promote yeast cell longevity, compared with the effects of treatment without the plant extracts/fractions (i.e., DMSO alone) (Figure 2). These data were assumed based on the yeast cell viability within the stationary phase on days 7 and 11, which indicated the chronological age of the yeast cells.

As previously reported, the survival of *S. pombe* cells that were grown in a standard glucose concentration of 3% decreased by day 7, and hardly any cells survived beyond day 10 [35]. Therefore, assessment of cell viability within 7 and 11 days may have allowed conceptual perspective on the aging process of *S. pombe*. It is worth noting that prolongation of the yeast lifespan was better after treatment with several extracts/fractions than after calorie restriction treatment; in particular, these were the water extract of *D. integrifolia* (10.000 ppm) and the chloroform fraction (2505 ppm) and water extract (10000 ppm) of *G. parviflora*. Therefore, these extracts/fractions have high potential properties as antiaging agents.

**Figure 3:**
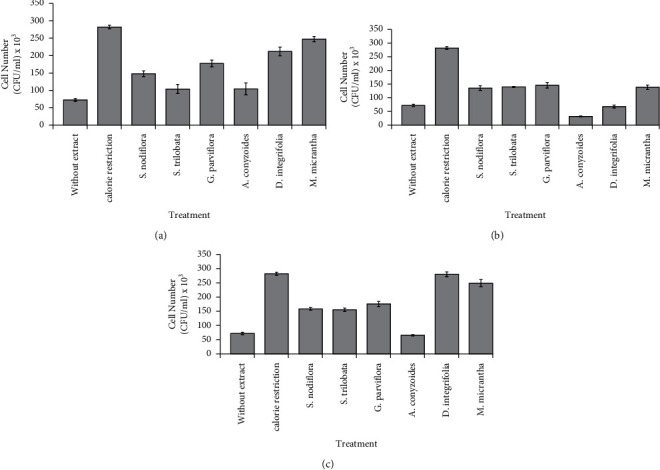
Effect of (a) water fraction, (b) chloroform fraction, and (c) water extract on the cell number of stationary phase-yeast cells (11 days of treatment incubation). Yeast cells were grown in YES medium containing an optimum concentration of plant extract/fractions as observed previously in oxidative stress tolerance assay. Yeast cells grown in EMM medium supplemented with DMSO were designated as calorie restriction for the positive control treatment, while yeast cells grown in YES medium supplemented with DMSO only without any extract/fractions addition were used as control. Each culture treatment was then incubated for 11 days and enumerated for cell concentration based on plate count assay using YES agar. Plates were incubated for three days at 30°C.

Further analysis of yeast cell survival on day 11 using quantitative plate count assay confirmed that all water fractions increased the survival of yeast cells, compared with the effects of control and treatment with DMSO only and without the extracts/fractions (Figure 3(a)). Moreover, treatment with the chloroform fractions of all plants, except *A. conyzoides*, significantly increased cell survival, compared with the effects of treatment without the extracts/fractions (Figure 3(b)). All the plant water extracts and fractions enhanced the survival rate of yeast cells, but *A. conyzoides* showed insignificant enhancement (Figure 3(c)). Unlike our findings in the qualitative spot assay, we noticed that only the water extract of *D. integrifolia* (10.000 ppm) increased the cell survival at a relatively similar value with that of the calorie restriction treatments (Figure 3(c)). This result suggested that treatment with the water extract of *D. integrifolia* mimicked calorie restriction-dependent cell longevity in nutrient-rich conditions. Similarly, previous studies revealed that treatment with green tea and resveratrol mimicked calorie restriction conditions, which induce longevity in yeast cells [36, 37].

Calorie restriction has been known to slow aging in model organisms and risk or degenerative diseases in mammalian cells [38]. Calorie restriction conditions (0.5% glucose) were shown to mediate longevity of the yeast *S. cerevisiae* grown by decreasing the growth factor signaling of Target of Rapamycin- (TOR-) Sch9 pathways and increasing Nicotinamide Adenine Dinucleotide- (NAD-) dependent deacetylase Sir2 and autophagy [24, 39, 40]. Moreover, reduced TOR signaling in both fission and budding yeasts was shown to enhance mitochondrial respiratory activity and coupling, which subsequently induce both Rim15-mediated ROS detoxification and adaptive mitochondrial ROS signaling, leading to lifespan extension [41]. Therefore, to clarify the involvement of the mitochondria in the mechanism of action of the plant extracts/fractions in extending yeast lifespan, we further tested the effect of the extracts/fractions on the mitochondrial membrane potential.

### 3.4. Effect of the Plant Extracts/Fractions on Mitochondrial Activity

**Figure 4:**
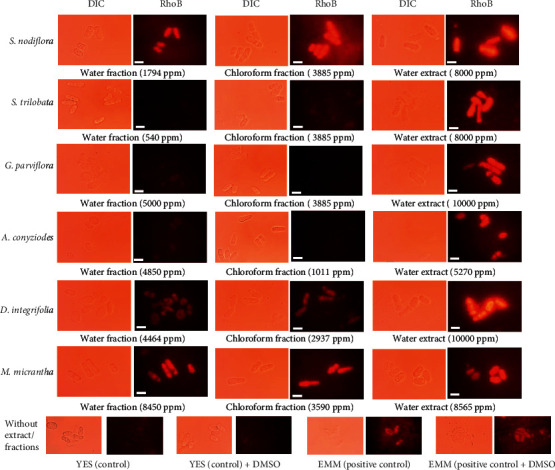
Effect of plant extract/fractions on the yeast mitochondrial activity. Yeast was grown in YES broth medium containing various concentrations of extract/fractions. Cells were then harvested and suspended with phosphate buffer. Mitochondrial activity was identified using Rhodamine 123 as a probe under fluorescence microscopy observation.

The mitochondria play important roles in regulating yeast chronological lifespan through adaptive mitochondrial ROS signaling [41]. Such phenomenon has been known to be conserved in yeast, nematode *Caenorhabditis elegans*, and mice [41–44]. As expected, treatment with the water extract of *D. integrifolia* significantly increased the mitochondrial membrane potential, as shown by the strong fluorescence intensity (Figure 4). Interestingly, in addition to the water extract of *D. integrifolia*, all water extracts of the plant samples induced mitochondrial activity (Figure 4). Such an increase in mitochondrial activity may further lead to adaptive mitochondrial ROS signaling, which can induce stress tolerance responses. Indeed, this was confirmed by the capability of the yeast cells treated with the water extract in combating strong H_2_O_2_-induced oxidative stress treatments (Table 3).

Interestingly, the water and chloroform fractions of *D. integrifolia*, *M. micrantha*, and *S. nodiflora* enhanced mitochondrial activity, similar to that seen with calorie restriction treatments. Although treatments with these water and chloroform fractions had shorter lifespan extension, compared with that of calorie restriction conditions, they were able to prolong the yeast lifespan, compared with the effect of treatment without the fractions. As reported by a previous study, mitochondria activity and biogenesis induce cell longevity in yeast [45]. Notably, not all water and chloroform fractions were found to induce mitochondrial activity, nevertheless to its potential antiaging activity. For example, the water and chloroform fractions of *A. conyzoides*, *G. parviflora*, and *S. trilobata* did not induce mitochondrial activity, but they promoted oxidative stress tolerance phenotype and slow aging (Figure 2). These results suggested that the development of oxidative stress tolerance and lifespan extension phenotype of yeast after the application of those particular fractions were independent of the adaptive mitochondrial ROS signaling.

Previous studies indicated that the oxidative stress tolerance of the yeast *S. pombe* was mediated by various pathways, including the stress-activated protein kinase and the cell integrity pathways [44], which mediate the activation of core environmental stress response (CESR) through the key transcription factors Sty1/Atf1 and Pap1 for strong and mild oxidative stress conditions, respectively [44, 46]. Therefore, we further confirmed the potential induction of CESR genes by the selected plant extracts/fractions.

### 3.5. Effect of *S. nodiflora* Extracts/Fractions on the Expression of the Gene Involved in Oxidative Stress Tolerance

Among the various genes in the CESR system, the key transcriptional factors gene pap1 and two genes of the downstream targets, including ctt1 and sod2, were analyzed in this study. These genes are essential for combating mild oxidative stress, which leads to adaptive oxidative stress response, direct ROS scavenging, and promotion of redox homeostasis in *S. pombe* [44, 46].

**Figure 5:**
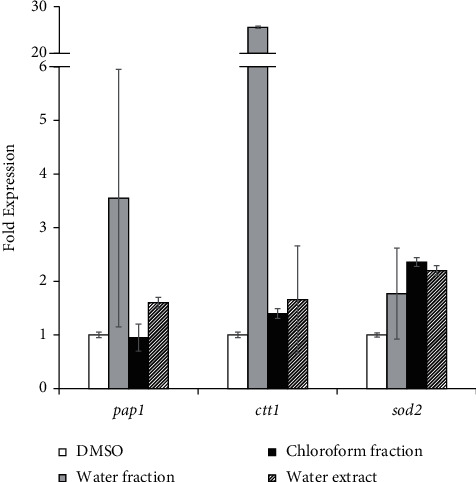
Antioxidant-related gene expression (pap1, ctt1, and sod2) of *S. pombe* cells treated with fraction and extract of *S. nodiflora* based on Reverse Transcriptase PCR analysis. Yeast cells were grown in YES liquid medium supplemented with water fraction, chloroform fraction, and water extract of *S. nodiflora* with each concentration of 1794, 3885, and 8000 ppm, respectively. *S. pombe* culture treated with DMSO was used as a control.

For gene expression analysis, we used the extracts and fractions of *S. nodiflora*. The fraction of *S. nodiflora* was shown to have quite strong in vitro antioxidant activity and strongly induced mitochondrial activity (Figure 4). The fraction and extract of *S. nodiflora* affected the transcriptional level of the target genes at different patterns. The water fractions increased the expression of all target genes, whereas ctt1 gene was found to be markedly increased 25-fold (Figure 5). The mRNA level of pap1 Transcriptional factor (TF) was increased by the water fraction and water extract, but no significant change in the pap1 mRNA level was detected following treatment with the chloroform fraction. The expression of sod2 gene was upregulated by chloroform fraction and water extract with an expression value of 2-fold compared to the DMSO control (Figure 5). These data indicated that primarily water fractions derived from *S. nodiflora* induced the pap1-dependent CESR systems in *S. pombe* likely by inducing mitochondrial activity. Such inductions of mitochondrial activity may develop an adaptive mitochondrial ROS signaling and induce the corresponding pap1-dependent CESR system. Indeed, the ctt1 gene encoding catalase protein was significantly transcribed following water fraction. As in yeast *S. pombe*, Ctt1 functions downstream of Tpx1-Pap1 TF and Sty1-Atf1 TF contributed to lifespan extension [47]. Ctt1 is required to protect the yeast cells from the toxicity of H_2_O_2_ radicals and the acquisition of oxidative stress resistance which are likely to increase following fraction-induced high mitochondrial activity [44]. In addition, the sod2 gene for the mitochondrial manganese superoxide dismutase was transcripted following chloroform fraction and water extract (Figure 5). As in yeast *S. cerevisiae*, Sod2 functions downstream of Sch9 contributed to lifespan extension [48]. Sod2p is required to protect mitochondrial DNA from the toxicity of superoxide anions that are likely to increase following extract-induced high mitochondrial activity [49]. Previous studies described that enhancement of the mitochondrial membrane potential in another yeast *S. cerevisiae* tor1Δ strain resulted in lifespan extension [41, 50]. A similar phenomenon was reported in a prior study, in which exogenous treatment with pyrrolo[1,2-a]pyrazine-1,4-dione that was extracted from marine bacterial culture with *Pseudomonas* sp. PTR-08 was shown to induce the longevity of the yeast *S. pombe*, likely by modulating the pap1-ctt1 pathway, in addition to the cell cycle processes [31].

### 3.6. LC-MS/MS Profile of *S. nodiflora* Fractions

**Figure 6:**
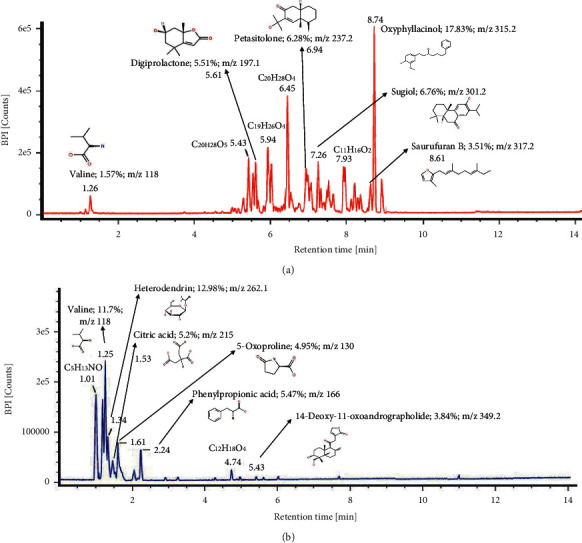
LC-MS/MS profile and predicted compounds of *S. nodiflora* fractions: (a) water fraction; (b) chloroform fraction.

Results from LC-MS/MS analysis showed that the water and chloroform fractions of *S. nodiflora* have more than 20 compounds. Nevertheless, we identified 6 predicted compounds from each fraction which were dominant and clearly identified as shown in Figures 6(a) and 6(b) (mass spectrum available on Supplementary Figures 1 and 2). The dominant compounds on water fractions, primarily oxyphyllacinol, valine, and sugiol, were reported as having strong antioxidant activity in vitro and in the cell line model [51–53]. Interestingly, sugiol was also reported as having numerous pharmacological properties including antimicrobial, anti-inflammatory, and antivirus along with anticarcinoma [53]. As for chloroform fractions, some compounds including 14-deoxy-11-oxoandrographolide, citric acid, and also valine exhibited antioxidant activity in vitro [54–56]. Of note, 14-deoxy-11-oxoandrographolide is a compound belonging to the andrographolide class (diterpenoids), which is also popular with other pharmaceuticals activities, that is, anti-inflammatory and antidiabetic [57, 58]. These results suggested that these dominant compounds especially in water fractions proven with strong in vitro and cell line antioxidant activity might affect the longevity in yeast cells. However, further research should be examined to elucidate each of those potential compounds and retest the corresponding effect in the yeast cells, since reports of the particular activity from those compounds in relation to antioxidant and antiaging mechanisms in yeast cells are still limited.

## 4. Conclusions

The water and chloroform extracts/fractions of Asteraceae plants can alter the cellular functions, induce an intracellular oxidative stress tolerance mechanism, and extend the lifespan of *S. pombe*. Mitochondrial activity increased after treatment with the extracts/fractions, which likely induced mitochondrial adaptive ROS signaling. These results would be essential for the development of the bioactive compounds of Asteraceae plants, particularly as pharmacologic antiaging/antioxidant products.

## Figures and Tables

**Table 1:** Species, local name, and voucher specimen number of all samples used in this study.

No.	Species	Local name	Voucher specimen number
1	*Ageratum conyzoides* L.	Bandotan	BMK0176092016
2	*Dichrocephala integrifolia* (L.f.) Kuntze	Jukut Meurit	BMK0406102018
3	*Galinsoga parviflora* (cav.)	Loseh	BMK0415102018
4	*Mikania micrantha* Kunth	Sembung Rambat	BMK0419012018
5	*Sphagneticola trilobata* L. Pruski	Wedelia/Pruski	BMK0364102018
6	*Synedrella nodiflora* L.	Jotang Kuda	BMK0446012018

**Table 2:** Primer pairs used in the analysis of gene expression by using qRT-PCR.

Targeted genes	Primer sequence
Reference gene (act1+)	
Forward (F)	5′ CGGTCGTGACTTGACTGACT 3′
Reverse (R)	5′ ATTTCACGTTCGGCGGTAGT 3′

Transcriptional factor Pap1 (pap1+)	
Forward (F)	5′ TGGATGGCGATGTTAAGCCT 3′
Reverse (R)	5′ GCAGCACGGTTTTGAGCTTT 3′

Superoxide dismutase 2 (sod2+)	
Forward (F)	5′ ATTTGGAGGGAGAGGTTGCC 3′
Reverse (R)	5′ GATTGATGTGACCACCGCCA 3′

Catalase (ctt1+)	
Forward (F)	5′ TCGTGACGGCCCTATGAATG 3′
Reverse (R)	5′ AGCAAGTGGTCGGATTGAGG 3′

**Table 3:** Effect of selected concentration of plant extract/fraction on yeast cells viability.

Plants	Extract/fraction^*∗*^	Selected potential concentration (ppm)	Concentration based on IC50^*∗∗*^	Cells viability against oxidative stress^*∗∗∗*^
*A. conyzoides*	Water fraction	4850	5X	++
	Chloroform fraction	1011	5X	−
	Water extract	5270	5X	+

*D. integrifolia*	Water fraction	4464	4X	+
	Chloroform fraction	2937	3X	++
	Water extract	10000	5X	+++

*G. parviflora*	Water fraction	5000	5X	+++
	Chloroform fraction	2505	5X	++
	Water extract	10000	5X	++

*M. micrantha*	Water fraction	8450	5X	++
	Chloroform fraction	3590	2X	++
	Water extract	8565	5X	++

*S. nodiflora*	Water fraction	1794	3X	+++
	Chloroform fraction	3885	3X	+++
	Water extract	8000	4X	++

*S. trilobata*	Water fraction	540	5X	++
	Chloroform fraction	846	3X	++
	Water extract	444	3X	++

Calorie restriction	Without extract/fractions	−	−	+
	DMSO			+

Control	Without extract/fractions	−	−	−
	DMSO			−

^*∗*^The IC50 value of water extract was applied based on a previous study. ^*∗∗*^IC50 concentration used was selected based on DPPH assay (Figure 1(a)). 
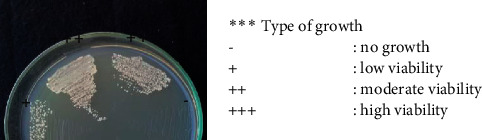
. 
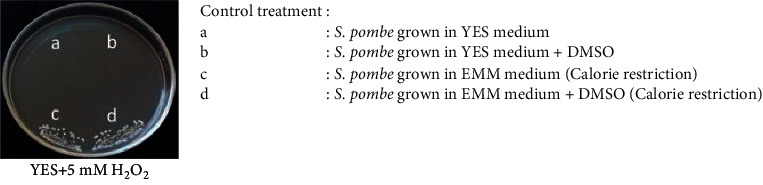
.

## Data Availability

All data analyzed during this study are included within the published article.

## Supplementary Materials

Supplementary Materials Mass spectrum of both *S. nodiflora* water and chloroform fractions.
