# Supplementary material for: Antiaging and Antioxidant Bioactivities of Asteraceae Plant Fractions on the Cellular Functions of the Yeast Schizosaccharomyces pombe
Source: Adv Pharmacol Pharm Sci. 2021 Sep 18;2021:2119634. doi: 10.1155/2021/2119634 (PMC8476265; doi:10.1155/2021/2119634)

Supplementary Material

Antiaging and antioxidant bioactivities of Asteraceae plant fractions on the cellular functions of the yeast *Schizosaccharomyces pombe.*

Rika Indri Astuti[1,4], Muhammad Eka Prastya[2], Irmanida Batubara[3,4*], Eka Budiarti[3], Aulia Ilmiyawati[3]

[1]*Department of Biology, Faculty of Mathematics and Natural Sciences, IPB University, IPB Dramaga Campus, Bogor, West Java 16680, Indonesia*

[2]*Research Center for Chemistry, Indonesian Institute of Sciences (LIPI), National Research and Innovation Agency (BRIN), Kawasan PUSPITEK, Serpong, Tangerang Selatan, 15314 Banten, Indonesia*

[3]*Department of Chemistry, Faculty of Mathematics and Natural Sciences, IPB University, IPB Dramaga Campus, Bogor, West Java 16680, Indonesia*

[4]*Tropical Biopharmaca Research Center, IPB University, Jl. Taman Kencana No. 3, Bogor, West Java 16128, Indonesia*

*Corresponding author: ime@apps.ipb.ac.id

This files includes:
Supplementary Figures S1 – S2

Supplementary Figure S1

Mass spectrum of 6 predicted dominant compounds on *S. nodiflora* water fractions

1. Digiprolactone

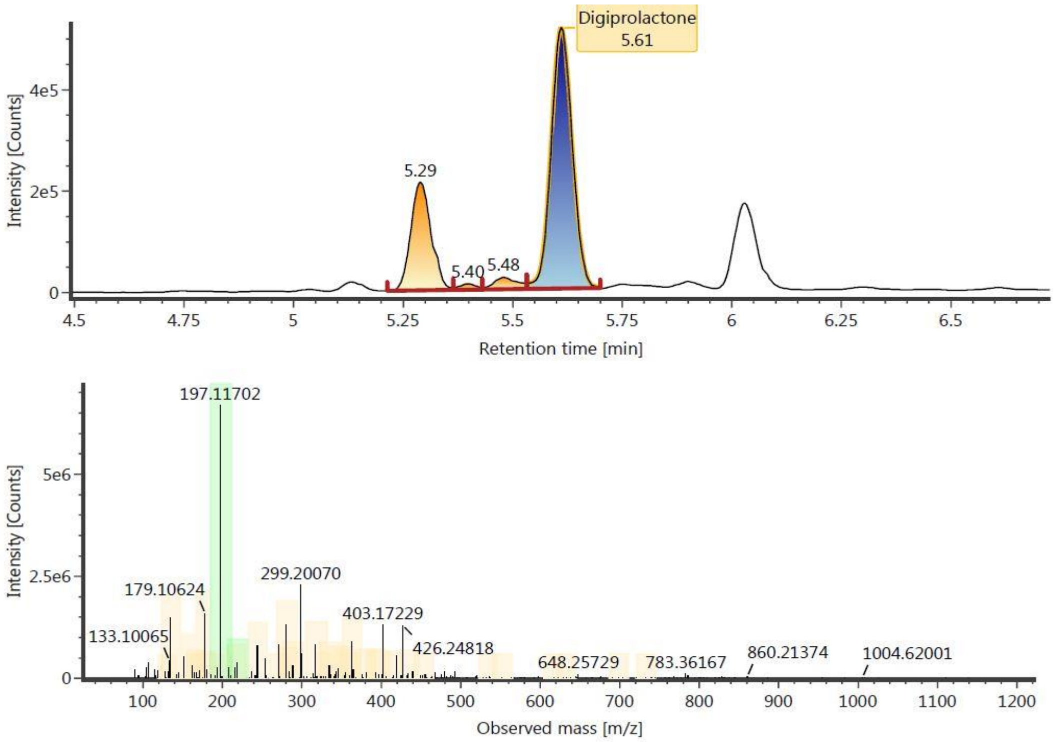

2. Oxyphyllacinol

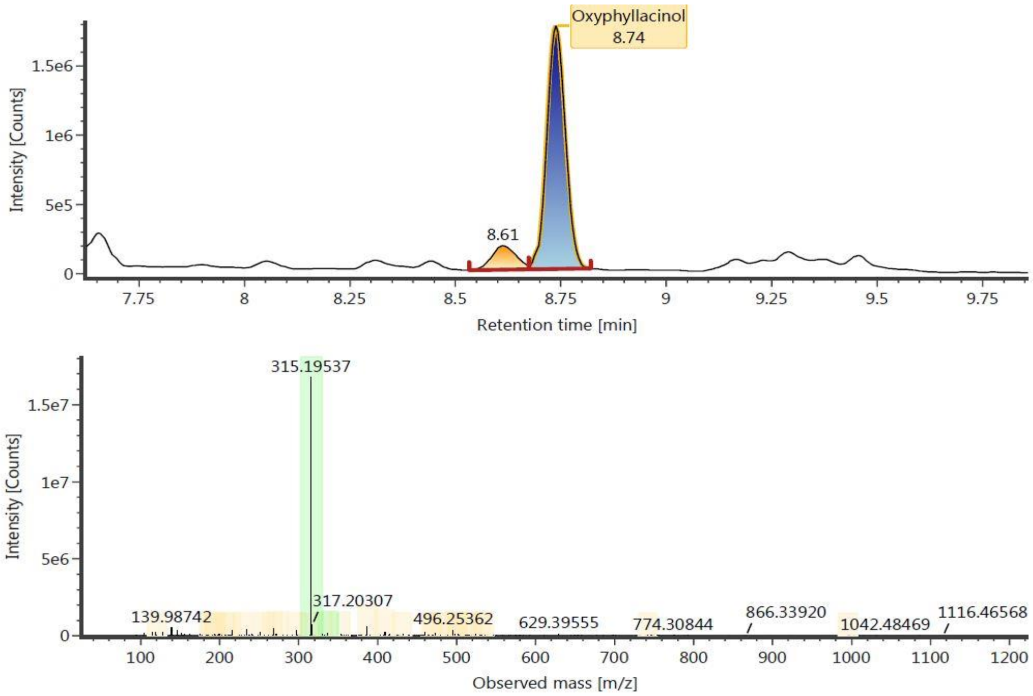

3. Petasitolone

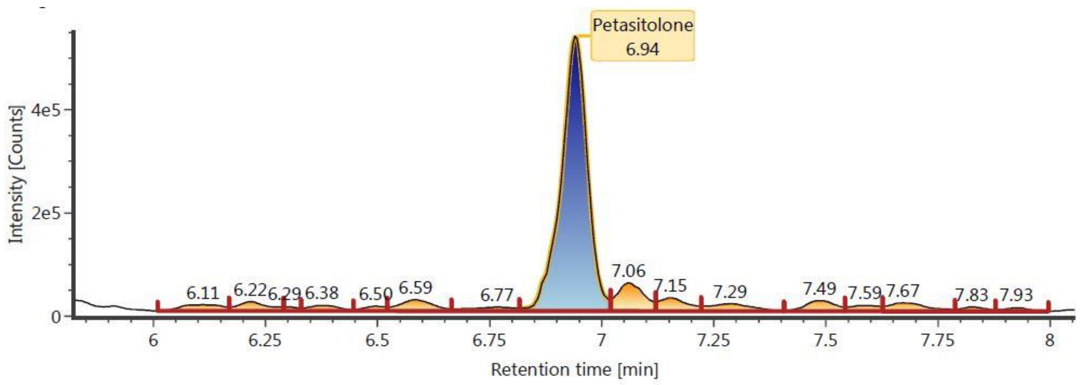

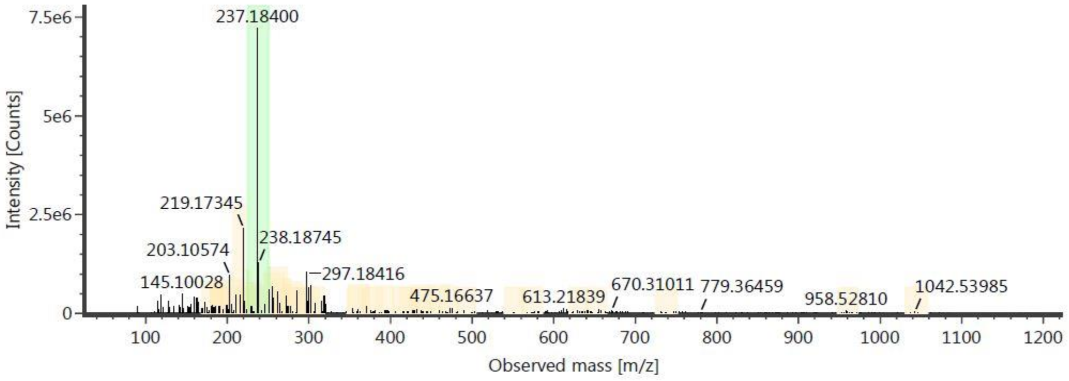

4. Saurufuran B

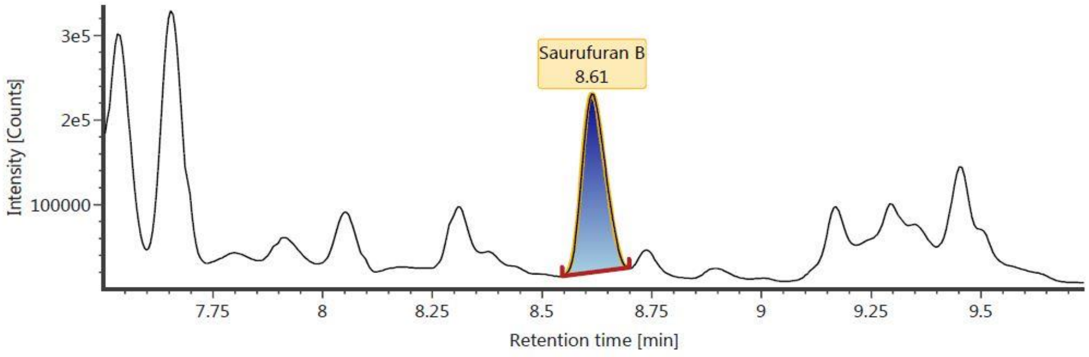

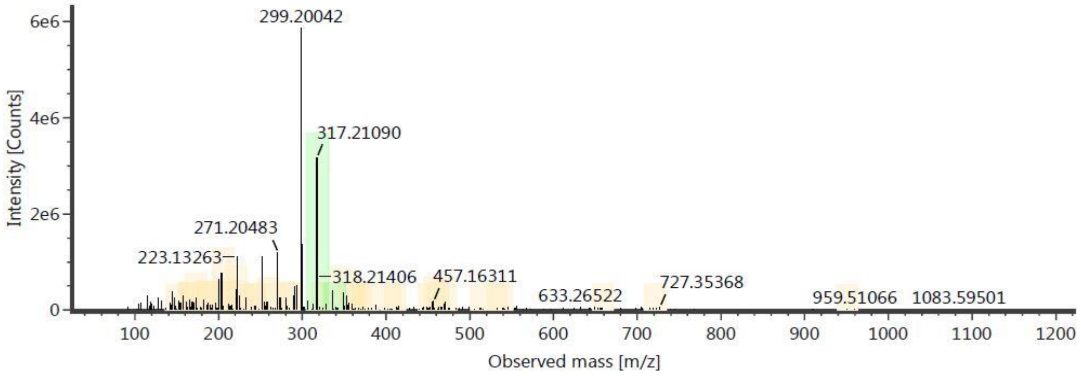

5. Sugiol

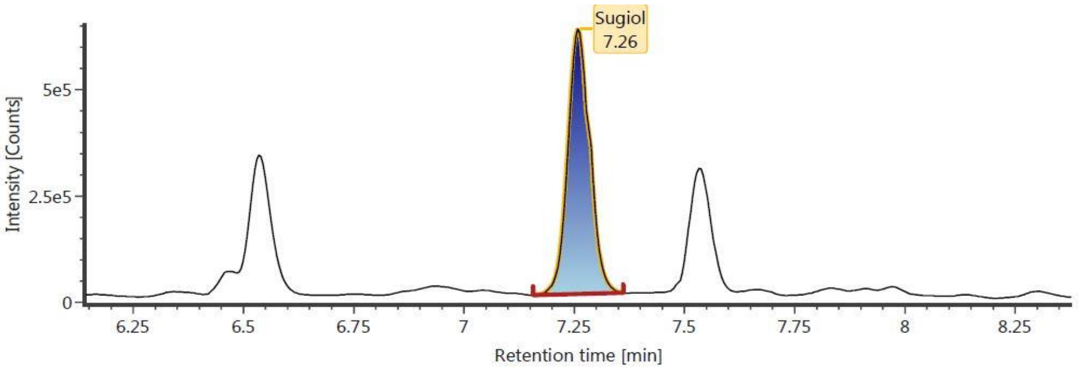

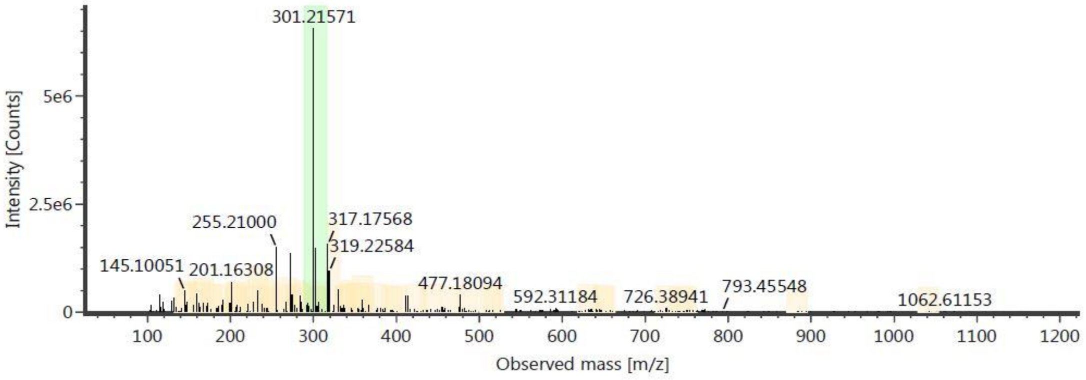

6. Valine

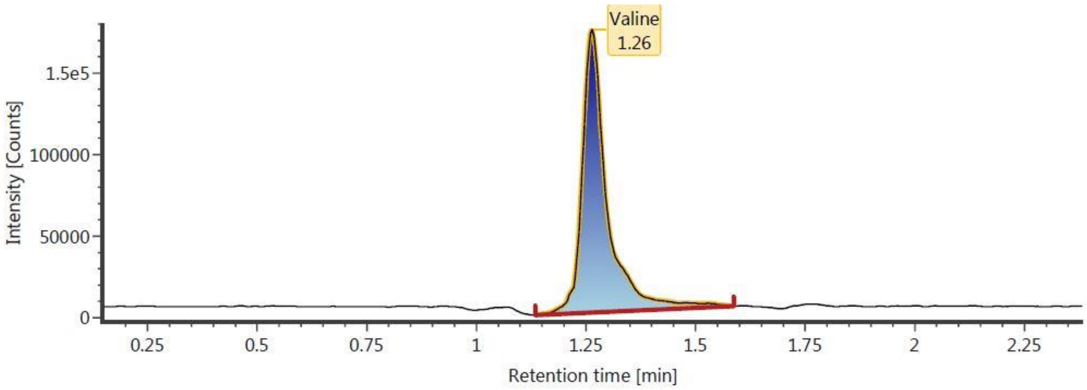

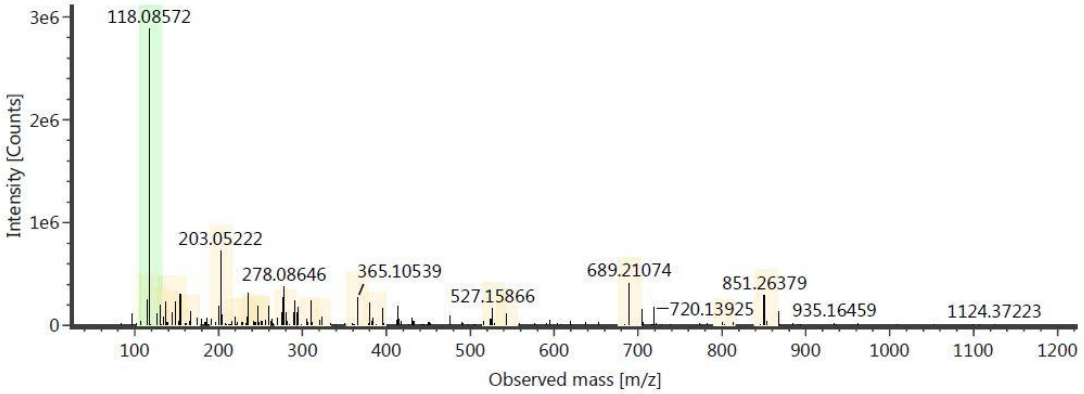

Supplementary Figure S2

Mass spectrum of 6 predicted dominant compounds on *S. nodiflora* chloroform fractions

1. 14-Deoxy-11-oxoandrographolide

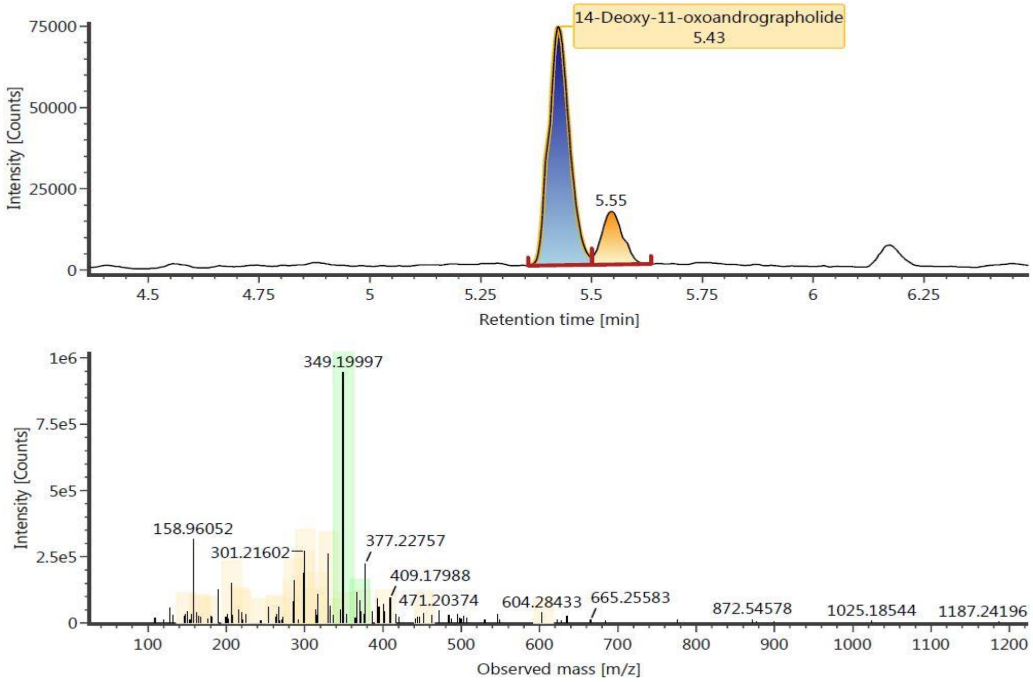

2. 5-Oxoproline

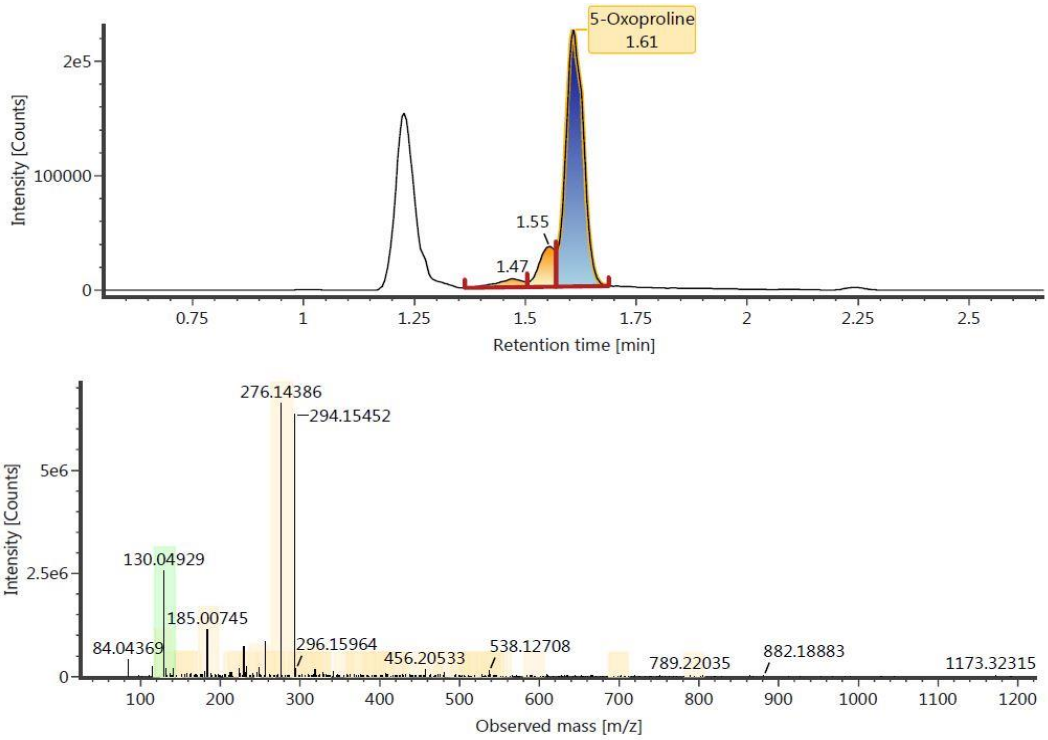

3. Citric acid

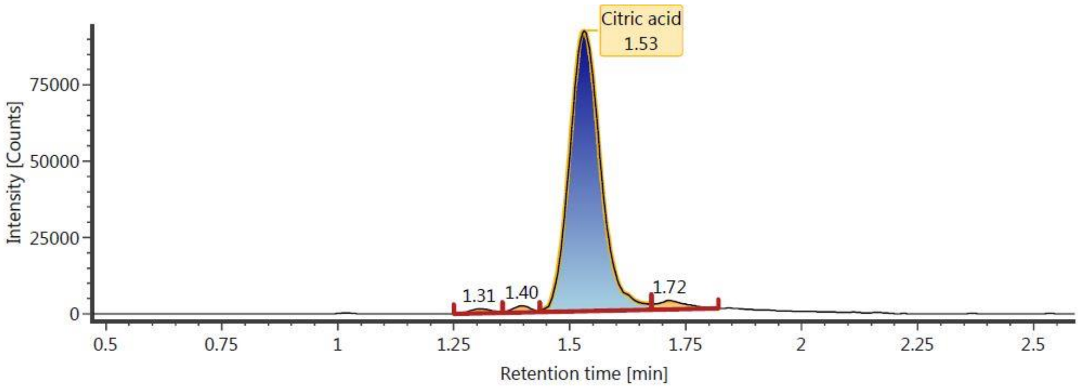

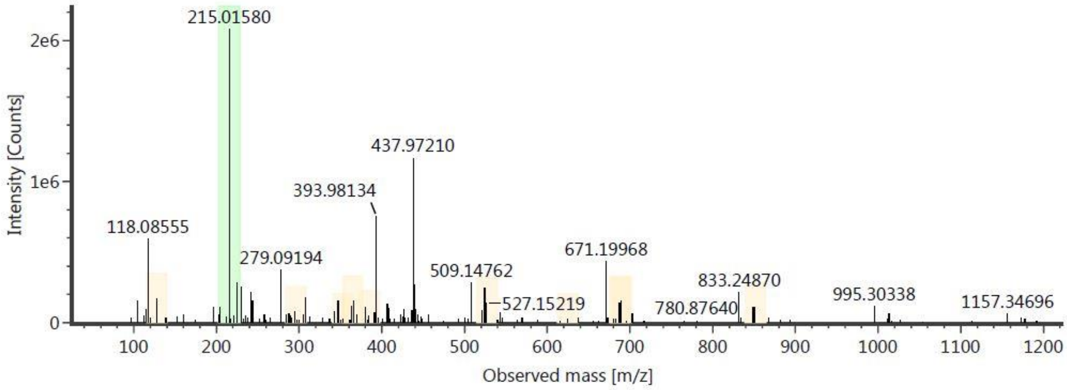

4. Valine

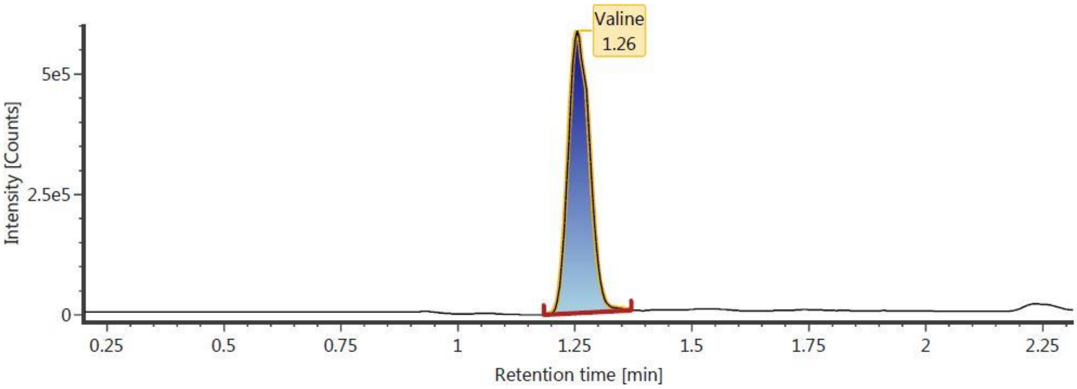

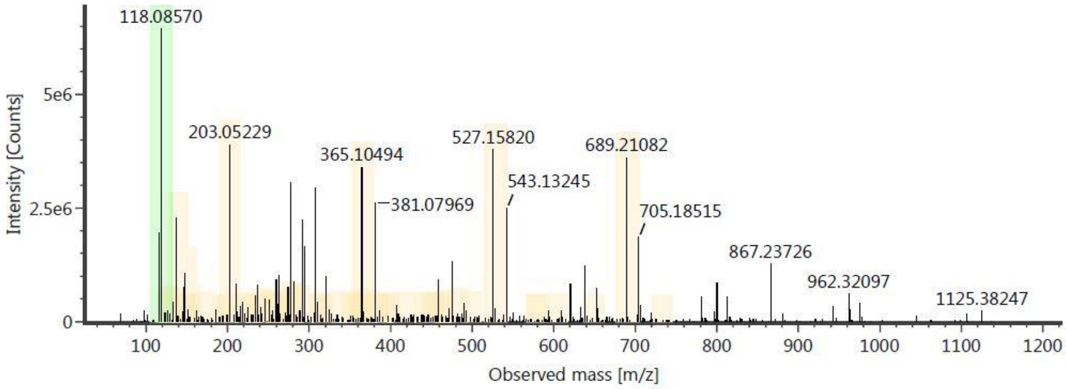

5. Heterodendrin

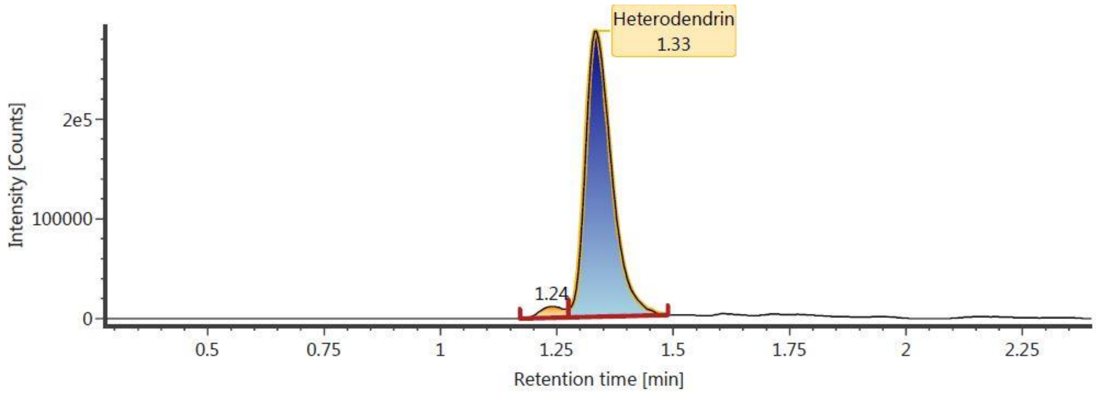

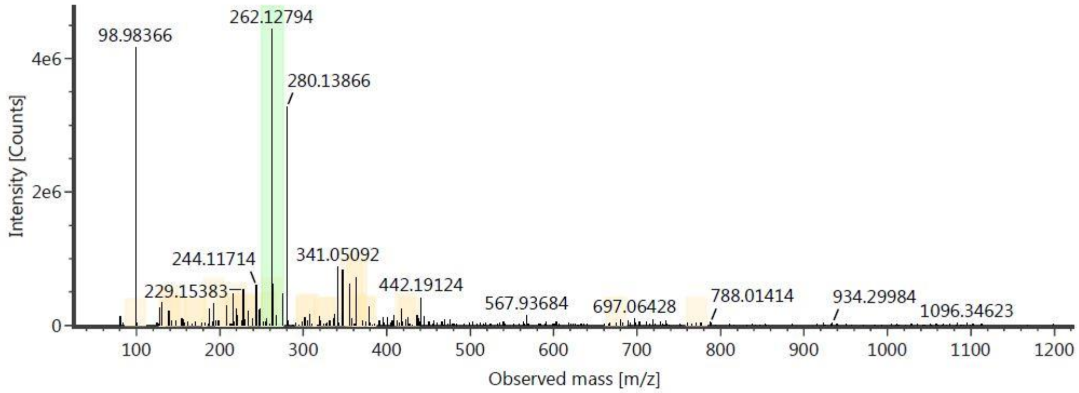

6. Phenylpropionic acid

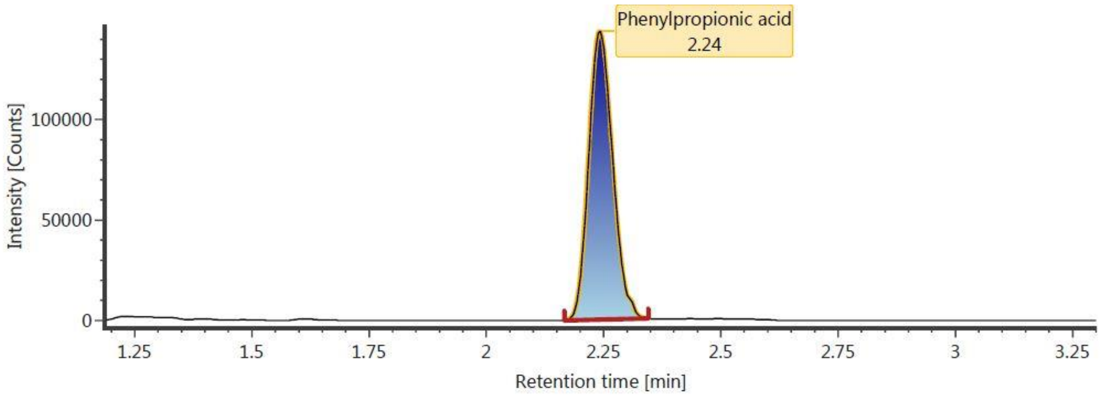

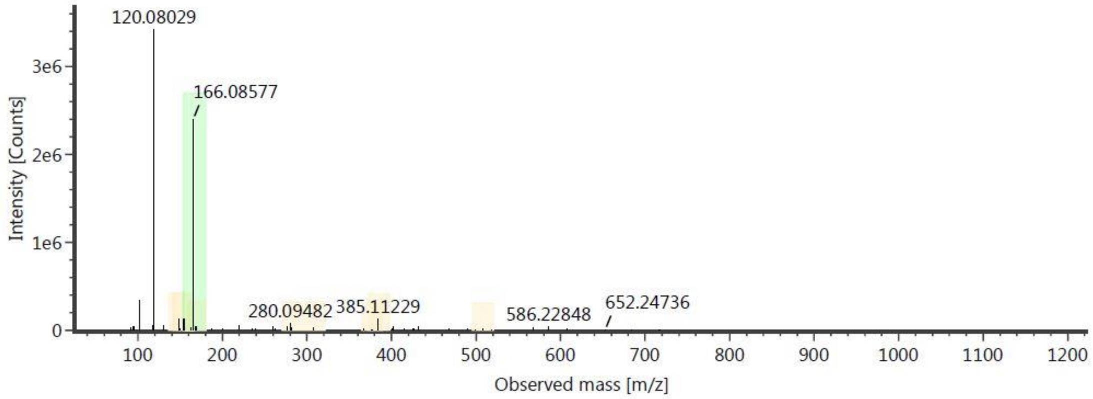

Supplement: Supplementary Materials — Mass spectrum of both S. nodiflora water and chloroform fractions. [file 2119634.f1.pdf]
